# MCFST: spatial domain identification method based on multi-view graph convolutional network and graph fusion network

**DOI:** 10.1093/bioinformatics/btag469

**Published:** 2026-07-03

**Authors:** Zilong Zhang, Hao Duan, Xin Gao

**Affiliations:** Computer, Electrical and Mathematical Sciences and Engineering (CEMSE), King Abdullah University of Science and Technology (KAUST), Thuwal 23955-6900, Kingdom of Saudi Arabia; School of Computer Science and Technology, Hainan University, Haikou 570228, China; Computer Science Program, Computer, Electrical and Mathematical Sciences and Engineering Division, King Abdullah University of Science and Technology (KAUST), Thuwal 23955-6900, Kingdom of Saudi Arabia; Center of Excellence for Smart Health (KCSH), King Abdullah University of Science and Technology (KAUST), Thuwal 23955-6900, Kingdom of Saudi Arabia; Syneron Opal, George Town 10281, Cayman Island

## Abstract

**Motivation:**

The emergence of spatial transcriptomics, which integrates spatial and gene expression information, has greatly advanced research in disease mechanisms and developmental biology. A core task in this field is spatial domain identification, which reveals regions with shared molecular signatures and histological features, thereby facilitating the study of tissue function and pathology. Although existing methods have achieved promising performance, many of them still face limitations in effectively integrating heterogeneous information from multiple views, such as gene expression, spatial coordinates, and spatially informed expression profiles. In particular, discrepancies across views may lead to inconsistent representations and distorted similarity relationships, which can reduce the accuracy and robustness of spatial domain recognition.

**Results:**

To address these limitations, we propose MCFST, a graph neural network framework that integrates multi-view graph convolution with a fusion module guided by mutual information maximization. By incorporating diverse views of spatial data and aligning their representations, MCFST effectively captures latent patterns and achieves robust domain recognition. We evaluated MCFST against state-of-the-art methods on two simulated datasets with varying sparsity and noise levels, as well as three real spatial transcriptomics datasets. Results show that MCFST consistently outperforms baselines in spatial domain identification, highlighting its robustness and efficiency. Moreover, spatially variable genes detected from MCFST-derived domains exhibited clear spatial expression patterns, further confirming the accuracy and utility of MCFST.

**Availability:**

The code implementation of the MCFST algorithm is publicly available at https://github.com/dw666666/MCFST.

## 1 Introduction

Spatial transcriptomics (ST) represents a novel technique that links gene expression data with spatial information, providing an important means to study cellular activities and intercellular interactions within tissue environments. This method makes it possible to detect gene expression patterns at spatial resolution, thereby facilitating a more comprehensive understanding of tissue organization and revealing the functional relationships that underlie various biological processes (Larsson *et al*. 2021, Longo *et al*. 2021, Marx 2021, Rao *et al*. 2021, Zhang *et al*. 2022, Duan *et al*. 2024). In recent years, spatial transcriptomics has advanced rapidly, with over fifty technologies introduced to date. These strategies are commonly divided into two main types: hybridization-based methods performed in situ and sequencing-oriented techniques, such as seqFISH (Lubeck *et al*. 2014, Shah *et al*. 2016), seqFISH+ (Eng *et al*. 2019), MERFISH (Chen *et al*. 2015, Moffitt *et al*. 2018), STARmap (Wang *et al*. 2018) and FISSEQ (Lee *et al*. 2014), and spatial transcriptomics (Ståhl *et al*. 2016), SLIDE-seq (Rodriques *et al*. 2019), SLIDE-seqV2 (Stickels *et al*. 2021), HDST (Vickovic *et al*. 2019) and 10X Visium (https://www.10xgenomics.com) and other in situ capture-based technologies. With the gradual maturation of spatial transcriptomics technology, it has been widely employed in numerous medical investigations and has been utilized to explore different categories of cancers (Karras *et al*. 2022, Liu *et al*. 2023), reveal the complex regulatory networks between different cell types and cellular heterogeneity in neuroscience and pathology (Carlberg *et al*. 2019, Ji *et al*. 2020, Peng *et al*. 2020, Zhang *et al*. 2022), and study the development and pathogenesis of vital organs (Chen *et al*. 2022, 2023, Cui *et al*. 2025). It can be seen that spatial transcriptomics will provide key support for the discovery and treatment of many diseases such as cancer (Li and Wang 2021).

While grouping cells with comparable expression patterns is standard in single-cell transcriptomics, spatial transcriptomics advances this by identifying spatial domains. By integrating spatial coordinates with gene expression, it partitions tissue areas and reveals structural and functional organization (Walker *et al*. 2022, Sun *et al*. 2024).

In recent years, numerous algorithms have been developed. For instance, SEDR extracts gene features with a deep autoencoder, embeds spatial information via variogram autoencoder, and jointly optimizes both for spatial domain recognition (Xu *et al*. 2024). BayesSpace adds a spatial prior to cluster adjacent spots, improving precision (Zhao *et al*. 2021). SpaGCN integrates expression, spatial coordinates, and histology for domain identification (Hu *et al*. 2021). STAGATE jointly incorporates spatial and expression data through an adaptive graph attention–based autoencoder, compressing features into a latent representation of tissue structure (Dong and Zhang 2022). GraphST employs graph neural networks with self-supervised contrastive learning to enhance domain delineation (Long *et al*. 2023). MNMST reformulates domain recognition as multilayer network clustering, learning low-dimensional embeddings for effective identification (Wang *et al*. 2024).

In the past few years, graph-based multi-view clustering has gained attention for partitioning data from multiple perspectives. Given that spatial transcriptomics combines gene expression and spatial context, multi-view models are well-suited. Accordingly, many graph neural network–based multi-view methods have emerged, effectively capturing tissue structures and improving accuracy. For example, Spatial-MGCN uses a multi-view graph convolutional encoder to jointly learn from expression and coordinates, thereby delineating tissue regions (Wang *et al*. 2023). SpaNCMG applies a neighborhood-complementary hybrid-view graph convolutional network for profiling (Si *et al*. 2024). Nonetheless, these methods face challenges in feature integration across views (Wang *et al*. 2015, Yin *et al*. 2019). Large discrepancies may cause view divergence, distorting similarity matrices and reducing model accuracy (Wang *et al*. 2022).

To this end, we propose MCFST, a unified multi-view graph neural network framework for accurate spatial domain identification in spatial transcriptomics. MCFST constructs complementary views from gene expression, spatial coordinates, and spatially informed expression profiles, and learns a unified latent representation through a multi-view graph convolutional fusion autoencoder. In this framework, the mutual information maximization module is designed to enhance representation consistency across different views and extract shared latent grouping signals, thereby alleviating view divergence. In contrast, the graph fusion component focuses on integrating complementary graph structures from multiple views to construct a consistency graph that better captures spatial and transcriptional relationships among spots. Together, these modules enable MCFST to effectively align heterogeneous information and improve the robustness of spatial domain recognition.

The main contributions of this study are summarized as follows. First, we develop a unified multi-view graph neural network framework for spatial domain identification, which jointly model’s gene expression, spatial coordinates, and spatially informed expression features. Second, we introduce a mutual information maximization module to reduce inconsistencies among view-specific representations and enhance the learning of shared latent structures. Third, we design a graph fusion component to integrate complementary graph information from different views and construct a consistency graph for more accurate domain delineation. Finally, we evaluate MCFST on three real spatial transcriptomics datasets and two simulated datasets with varying sparsity and noise levels. Experimental results and ablation studies demonstrate that MCFST consistently outperforms state-of-the-art methods in spatial domain identification and spatially variable gene detection, enabling a more refined dissection of complex tissue architectures.

## 2 Results

### 2.1 Overview of the MCFST

**Figure 1:**
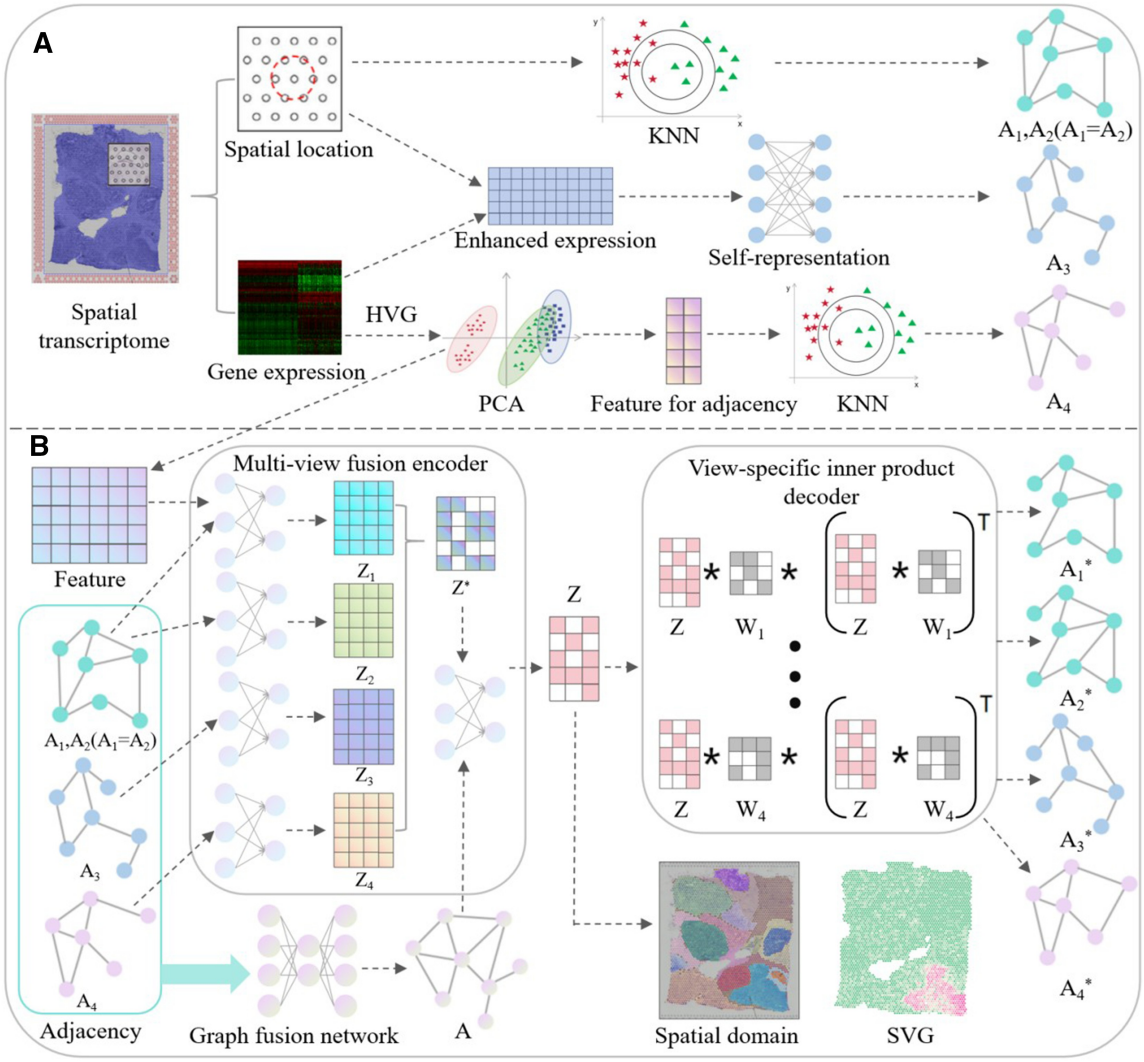
Overview of MCFST. (A) Feature extraction and multi-view construction. MCFST takes the gene expression matrix and spatial coordinates as inputs, derives spot-level features from expression data, and generates spatially enhanced profiles by integrating positional information. It then builds four adjacency matrices: two based on spatial information, one hybrid, and one from gene expression. (B) Latent feature learning and analysis. A multi-view graph convolutional autoencoder with mutual information maximization learns common latent representations from the four views and the coherence graph. These latent features are subsequently applied to spatial domain recognition and spatially variable gene detection.

MCFST is a multi-view graph neural network framework designed to perform spatial domain recognition and resolve biological organizational structures. It extracts highly variable genes from spatial transcriptomic data and applies preprocessing, principal component analysis (PCA) for dimensionality reduction to obtain feature representations for each spot (Fig. 1A). Moreover, MCFST uses spatial coordinate information to construct two identical spatial neighborhood matrices by KNN nearest neighbor algorithm, constructs a hybrid neighborhood matrix using spatially enhanced gene expression data by self-representation learning, and constructs a gene expression neighborhood matrix using the expression data of highly variable genes sequentially by PCA dimensionality reduction and KNN nearest neighbor algorithm (Fig. 1A).

Next, the neighborhood matrices of the four views are fused into a consistency graph using a graph fusion network, and the resulting spot features, the four views and the consistency graph are fed into a multi-view graph convolutional fusion autoencoder, where a mutual information maximization strategy is applied to obtain a shared latent representation (Fig. 1B). The obtained latent representation can be applied to tasks such as spatial domain recognition and the identification of spatially variable genes.

### 2.2 MCFST shows high robustness on simulated data

**Figure 2:**
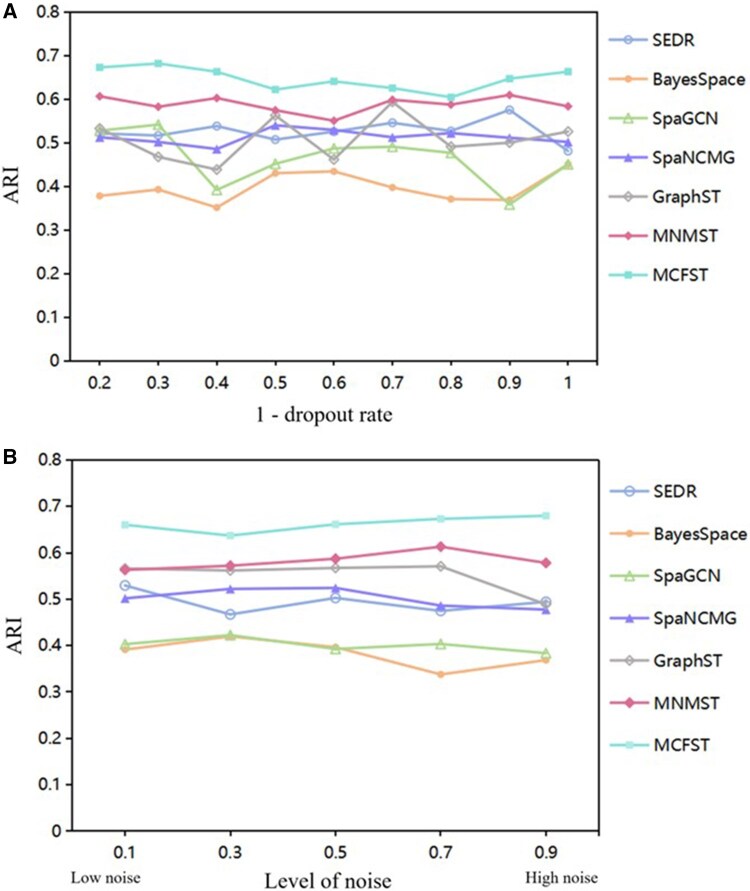
Comparison of MCFST with state-of-the-art approaches on simulated datasets. (A) Evaluation on datasets constructed with different sparsity degrees of the gene expression matrix. The horizontal axis shows the sparsity ratio of the matrix, while the vertical axis reports ARI values, reflecting the accuracy of spatial domain identification. (B) Assessment on simulated datasets perturbed by varying noise levels. The x-axis denotes the amount of noise added to the gene expression matrix, whereas the y-axis presents ARI scores, which serve as indicators of the reliability and quality of spatial domain recognition results.

To assess the robustness of MCFST under different data characteristics, we simulated spatial transcriptomic datasets by varying gene expression sparsity and noise levels. We then applied MCFST, GraphST, MNMST, BayesSpace, and SpaGCN to evaluate spatial domain recognition performance. Results show that MCFST and MNMST maintain stable accuracy across different sparsity levels, while BayesSpace, SpaGCN, and GraphST exhibit greater fluctuations. Notably, MCFST consistently outperforms all baselines in domain recognition under varying sparsity (Fig. 2A). When noise interference is introduced, both MCFST and GraphST demonstrate relatively stronger robustness, with MCFST again achieving the best performance (Fig. 2B). Overall, MCFST exhibits consistently superior robustness across diverse conditions.

### 2.3 MCFST performs precise profiling of human breast cancer tissue

**Figure 3:**
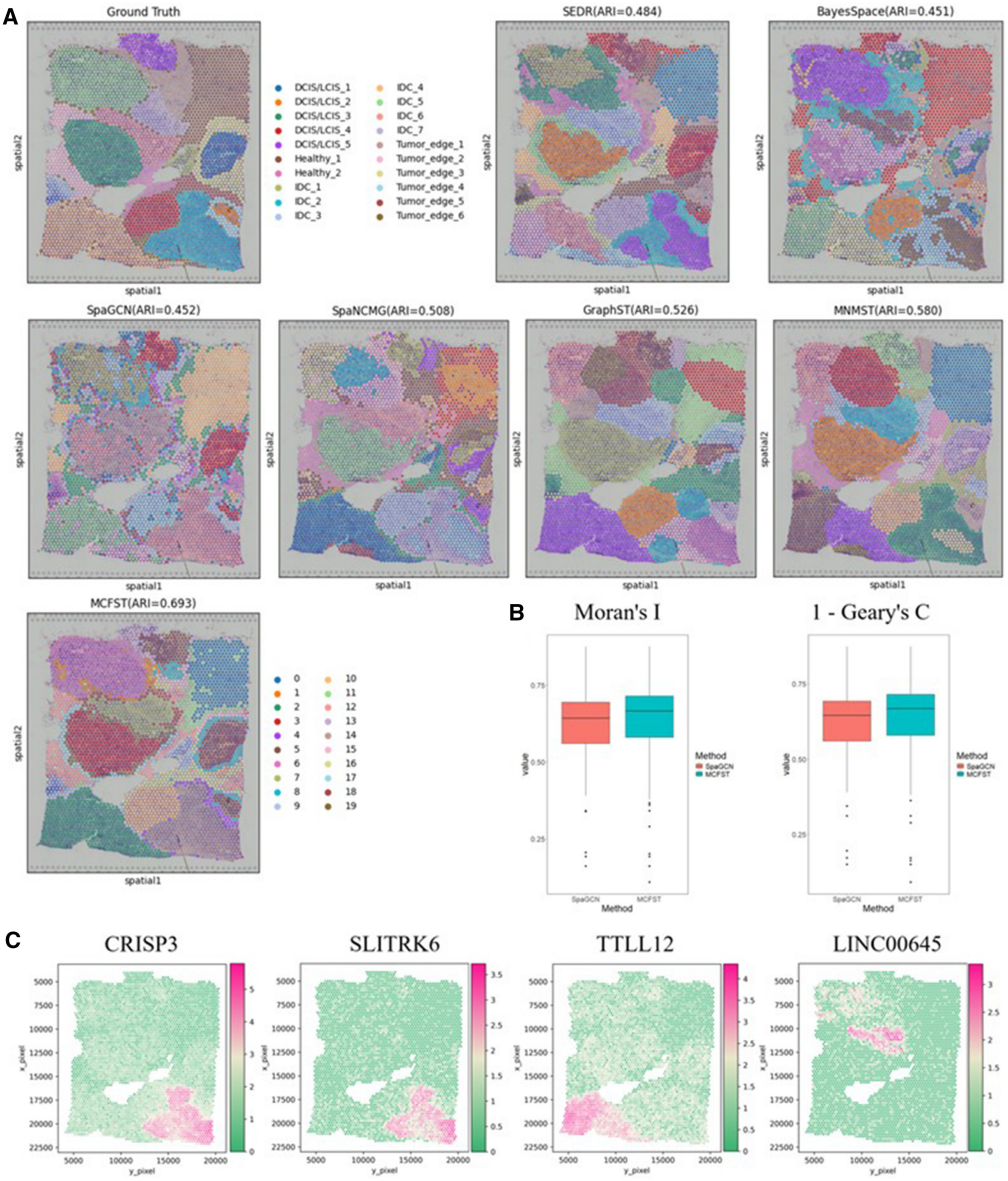
Spatial domain and SVG identification results on human breast cancer tissue. (A) Manual annotation and domain delineation by SEDR, BayesSpace, SpaGCN, SpaNCMG, GraphST, MNMST, and MCFST. (B) Boxplots of Moran’s I and 1−Geary’s C statistics for SVGs identified by MCFST and SpaGCN; boxes indicate interquartile ranges with medians, whiskers extend to 1.5× IQR, and outliers are shown as points. (C) Spatial expression patterns of representative SVGs (CRISP3, SLITRK6, TTLL12, LINC00645) identified by MCFST.

To evaluate MCFST in spatial domain recognition, we used a curated human breast cancer dataset containing four major tissue types—ductal/lobular carcinoma in situ (DCIS/LCIS), normal tissue, invasive ductal carcinoma (IDC), and tumor margin—further subdivided into 20 annotated subregions. MCFST was compared with SEDR, BayesSpace, SpaGCN, SpaNCMG, GraphST, and MNMST using the Adjusted Rand Index (ARI). MCFST achieved the best performance (ARI = 0.693), surpassing MNMST (0.58) and all other methods (<0.55) (Fig. 3A). Visualization further shows that MCFST delineates regions more precisely, e.g. IDC_4 (lower-left) and IDC_2 (lower-right), underscoring its advantage in fine-grained profiling.

We next assessed spatially variable genes (SVGs) based on MCFST and SpaGCN results. MCFST identified 229 SVGs, more than SpaGCN’s 180, with higher spatial autocorrelation: median Moran’s I (0.666 vs. 0.642) and median 1−Geary’s C (0.667 vs. 0.645) (Fig. 3B). Representative SVGs such as CRISP3 and SLITRK6 detected by MCFST displayed more pronounced spatial expression patterns, aligning with structural regions like IDC_2 and IDC_4 (Fig. 3C). These findings highlight MCFST’s superior performance in both spatial domain recognition and SVG detection.

### 2.4 MCFST provides precise identification of hierarchical structures in the human dorsolateral prefrontal cortex

In addition to profiling breast cancer tissues, we validated MCFST on normal human tissues using the human dorsolateral prefrontal cortex (DLPFC) dataset, a widely used benchmark on the 10X Visium platform. The dataset comprises 12 brain slices, each containing four to six cortical layers plus white matter, with expert annotations based on morphology and auxiliary criteria providing reliable ground truth.

**Figure 4:**
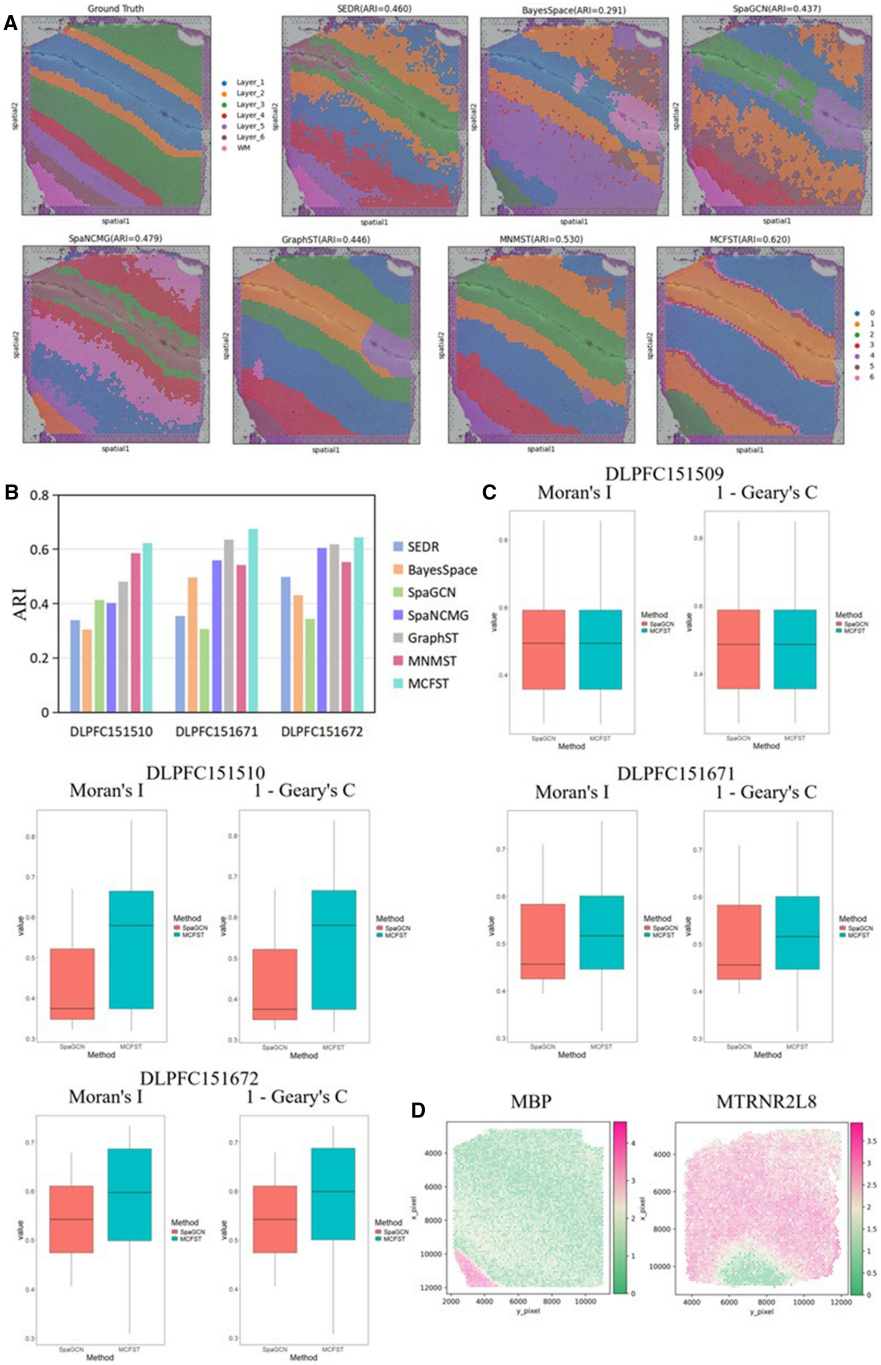
Results of spatial domain recognition and detection of spatially variable genes obtained by MCFST and other state-of-the-art comparison methods on the human dorsolateral prefrontal cortex (DLPFC) tissue dataset. (A) Spatial domains in DLPFC slice 151509 manually annotated and detected by SEDR, BayesSpace, SpaGCN, SpaNCMG, GraphST, MNMST, and MCFST. (B) Performance comparison of these methods on DLPFC slices 151510, 151671, and 151672, with the *x*-axis indicating slice IDs and the *y*-axis showing ARI scores for spatial domain accuracy. (C) Box plots of Moran’s I and 1-Geary’s C values for SVGs detected by MCFST and SpaGCN across four DLPFC slices (151509, 151510, 151671, 151672). In the boxplot, the hinges represent the first and third quartiles, while the line in the middle marks the median value. The whiskers extend to the most extreme observations that lie within 1.5 times the interquartile range, and any data points beyond this interval are depicted individually as outliers. (D) Spatial expression patterns of SVGs MBP and MTRNR2L8 identified by MCFST.

We compared MCFST with SEDR, BayesSpace, SpaGCN, SpaNCMG, GraphST, and MNMST on four slices (DLPFC1,51,509 DLPFC151510, DLPFC151671, DLPFC151672). MCFST achieved the best accuracy, with ARI scores of 0.620, 0.622, 0.676, and 0.645, significantly outperforming all baselines (Fig. 4A and B). The spatial partitions produced by MCFST closely matched the hierarchical cortical organization, with only minor misclassified points. MNMST and GraphST ranked next, typically scoring above 0.5 ARI, while other methods performed poorly and often misclassified spots, leading to confused domain boundaries. These results confirm that MCFST offers clear advantages in mapping the spatial organization of normal tissues.

We further applied MCFST to detect spatially variable genes (SVGs) across the same four slices, identifying 13, 13, 8, and 11 SVGs, respectively—considerably more than SpaGCN, which detected only 13, 3, 3, and 2. In addition, MCFST achieved comparable Moran’s I and 1−Geary’s C values to SpaGCN on DLPFC151509, and significantly higher values on the other three slices, e.g. DLPFC151510 (Fig. 4C). These findings demonstrate MCFST’s superior accuracy and robustness in both spatial domain recognition and SVG detection on normal human tissues.

What’s more, considering the precision of the spatial domain recognition outcomes, spatially variable genes such as MBP identified by MCFST in section DLPFC151509 and MTRNR2L8 identified in section DLPFC151672 are in good agreement with the structure of the tissue stratification (Fig. 4D). Thus, MCFST can also better identify spatially variable genes in human dorsolateral prefrontal cortex tissue.

### 2.5 MCFST improves recognition of complex anterior mouse brain tissue

**Figure 5:**
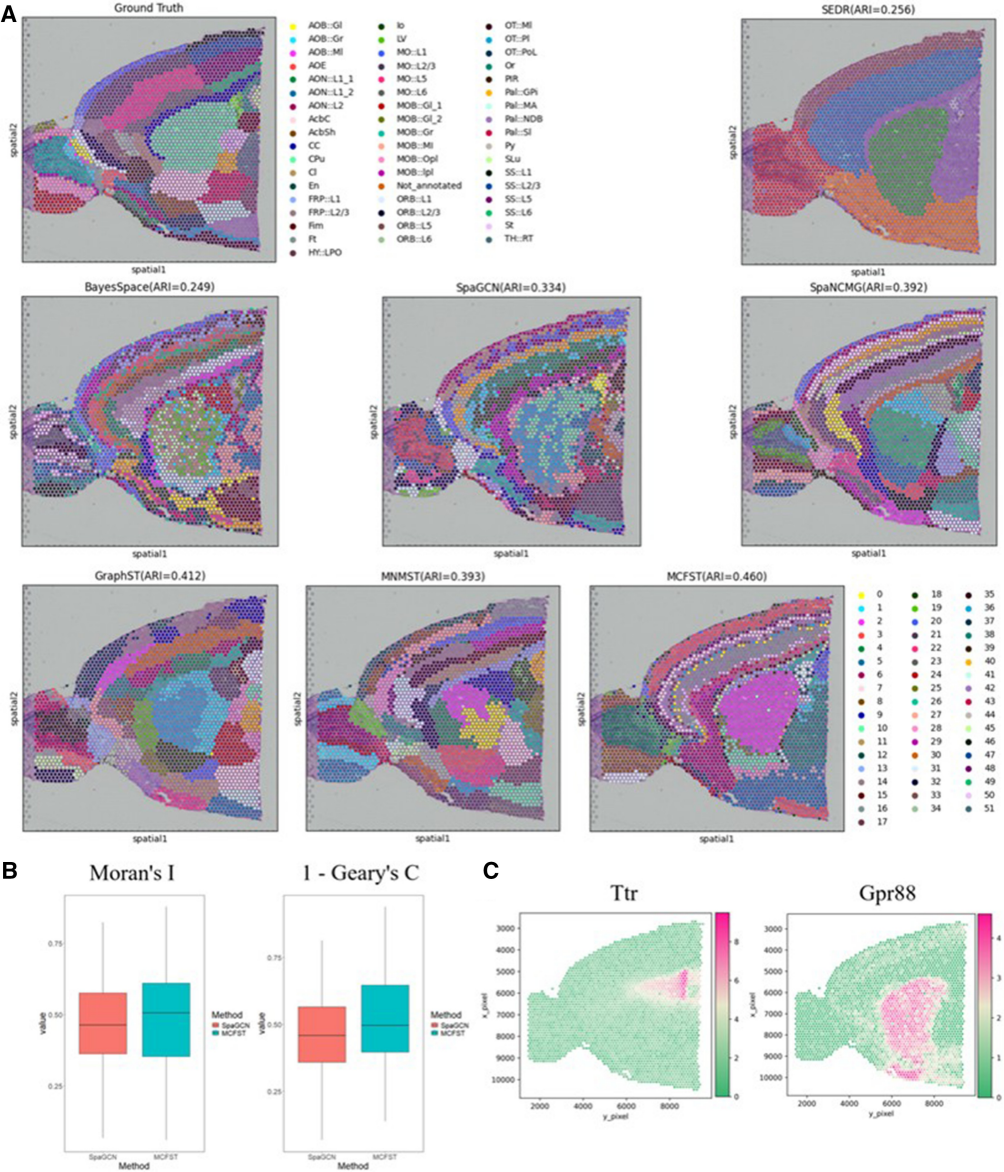
Results of spatial domain recognition and spatially variable gene detection obtained by MCFST and state-of-the-art comparison methods on the mouse anterior brain tissue section dataset. (A) Spatial domains delineated and detected by SEDR, BayesSpace, SpaGCN, SpaNCMG, GraphST, MNMST, and MCFST on the manually annotated mouse anterior brain tissue section dataset. (B) Box line plots of Moran’s I values and 1-Geary’s C values for spatially variable genes detected by MCFST and SpaGCN in the mouse anterior brain tissue section dataset. In a boxplot, the lower and upper hinges correspond to the first and third quartiles, and the line in the middle marks the median. The whiskers extend outward from the hinges to the most extreme observations lying within 1.5 times the interquartile range, whereas values that go beyond this limit are plotted individually and treated as outliers. (C) Spatial distribution patterns of the spatially variable genes Ttr and Gpr88 as identified by MCFST.

In addition, we examined a dataset consisting of mouse anterior brain tissue sections characterized by complex structural features, which was manually annotated to show that it contained 52 spatial domain divisions (Fig. 5A). By using spatial domain recognition algorithms such as MCFST on this mouse anterior brain tissue, it is possible to demonstrate the ability of the methods to profile tissues with fine structures. Compared with the advanced comparison methods, MCFST showed superior performance in terms of ARI metrics, with an ARI value of 0.46 for the spatial domain recognition results obtained. Except for GraphST, which performed relatively well, the ARI values of the other methods were below 0.4, which is a significant gap with the performance of our method MCFST. To more intuitively display the consistency between the detected regions and the manual annotations of tissue slices, we analyzed the visualization results produced by various spatial domain recognition approaches. These analyses showed that SEDR was unable to precisely capture or delineate the complex structural organization present in the anterior regions of the mouse brain tissue slices, while BayesSpace and SpaGCN produced recognition results containing a substantial number of anomalies. In contrast, the remaining methods were able to deliver relatively clearer delineation of the regions, aligning more closely with specific hierarchical structures (Fig. 5A). In contrast, the spatial domains detected by MCFST showed a higher degree of consistency with the manual annotations, especially in the tissue slice areas located at the right-middle and the far-left regions (Fig. 5A). These findings demonstrate that MCFST achieves superior accuracy in spatial domain detection relative to other methods. In addition, MCFST detected 470 spatially variable genes and SpaGCN detected 414 spatially variable genes, and MCFST showed clear superiority over SpaGCN both in the number of identified SVGs and in the statistical measures of SVGs, including Moran’s I and 1-Geary’s C values (Fig. 5B).

Moreover, SVGs such as Ttr and Gpr88 detected by MCFST have distinct spatial expression patterns (Fig. 5C).

### 2.6 Ablation study

**Figure 6:**
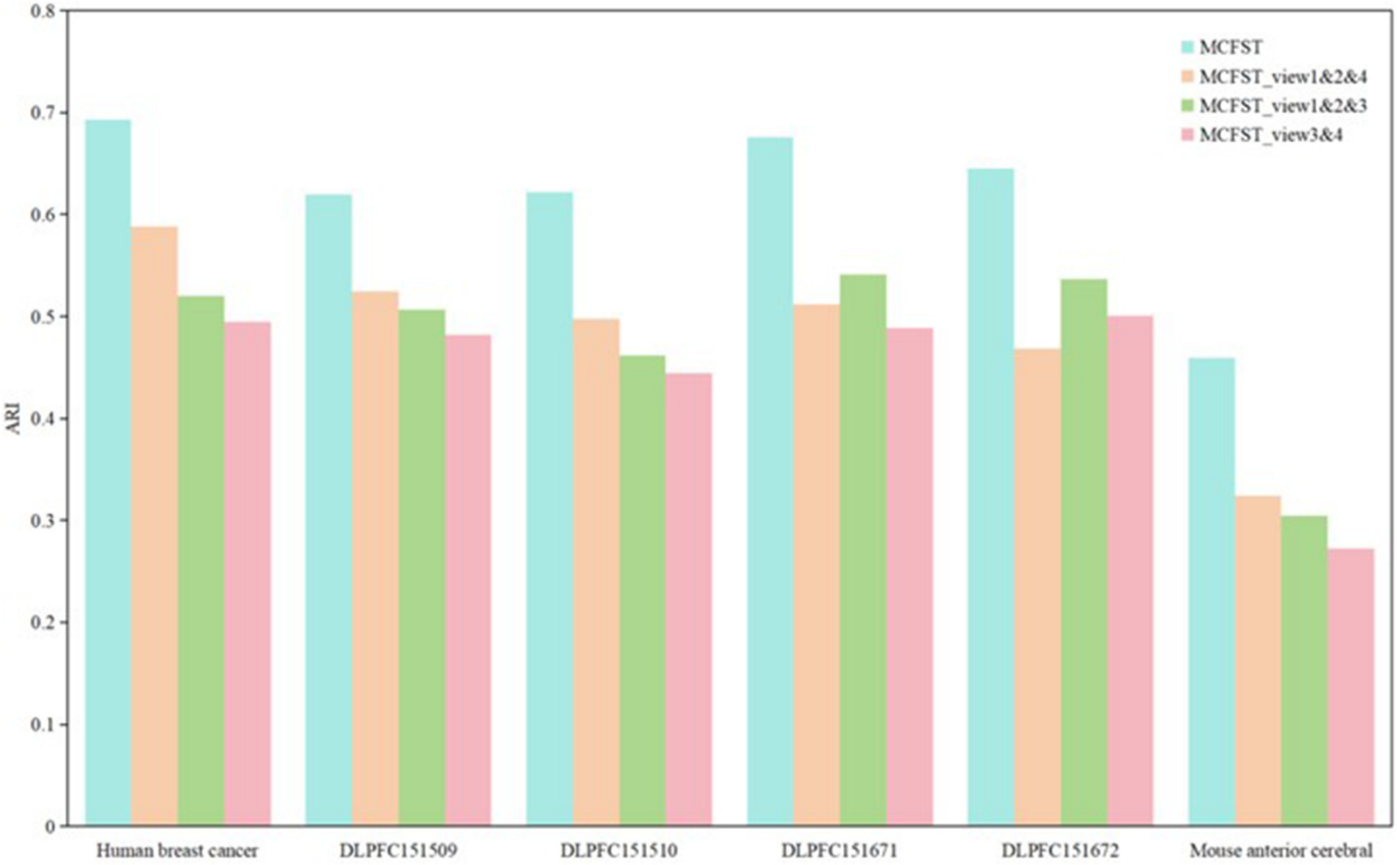
Performance comparison of MCFST and its variants across three datasets. MCFST is compared with three configurations (MCFST_view1&2&4, MCFST_view1&2&3, MCFST_view3&4) on the human breast cancer, human dorsolateral prefrontal cortex (DLPFC), and mouse anterior brain datasets. The x-axis denotes datasets, and the y-axis shows ARI scores for spatial domain recognition accuracy.

MCFST simultaneously incorporates spatially enhanced gene expression views, raw gene expression information views, and spatial coordinate views, which may introduce redundancy. Therefore, variants of MCFST were designed by removing the spatially enhanced gene expression data view (MCFST_view1&2&4), removing the gene expression information view (MCFST_view1&2&3), and removing the spatial information view (MCFST_view3&4). Furthermore, ablation experiments were conducted using MCFST_view1&2&4, MCFST_view1&2&3, and MCFST_view3&4 on real spatial transcriptomics datasets such as the human breast cancer tissue dataset. Ablation results demonstrate that MCFST consistently outperforms its three variants in spatial domain identification across all datasets (Fig. 6), confirming the effectiveness of the proposed framework.

In addition to removing individual views, we further performed module-level ablation experiments to assess the contributions of the mutual information maximization module and the graph fusion network. Specifically, we designed three additional variants: MCFST w/o MI, in which the mutual information maximization module was removed; MCFST w/o GFN, in which the graph fusion network was removed; and MCFST w/o MI+GFN, in which both modules were removed simultaneously. As shown in Table 1, the complete MCFST model achieved the highest ARI values across all six datasets. Removing the mutual information maximization module led to a decrease in performance, suggesting that this module contributes to cross-view representation consistency and helps alleviate view divergence. The removal of the graph fusion network resulted in a more pronounced decline, indicating that the learned consistency graph plays an important role in integrating complementary graph structures from different views. Furthermore, the simultaneous removal of both modules caused the largest performance degradation, further confirming their joint contribution to robust spatial domain recognition. Overall, these ablation results demonstrate that both the multi-view design and the proposed key modules are essential for the effectiveness of MCFST.

**Table 1:** Module-level ablation results of MCFST on six spatial transcriptomics datasets.

Dataset	MCFST	MCFST w/o MI	MCFST w/o GFN	MCFST w/o MI+GFN
Human breast cancer	0.691	0.656	0.623	0.597
DLPFC151509	0.618	0.586	0.554	0.533
DLPFC151510	0.620	0.583	0.551	0.518
DLPFC151671	0.674	0.636	0.602	0.561
DLPFC151672	0.642	0.604	0.572	0.546
Mouse anterior cerebral	0.457	0.421	0.389	0.356

## 3 Discussion

By integrating spatial information with traditional transcriptomics, spatial transcriptomics enables the localization of gene expression, generating detailed maps across tissue regions and structures. This supports key tasks such as spatial domain recognition and identification of spatially variable genes (SVGs). Accurate delineation of spatial domains, together with reliable detection of spatially expressed genes, is essential for understanding tissue organization and biological function.

We introduce MCFST, a spatial domain identification framework that extends multi-view graph convolutional networks with a graph fusion module and a mutual information maximization strategy. MCFST constructs multiple views (e.g. spatial location, gene expression), learns potential representations from each view, and integrates them consistently, enabling both spatial domain recognition and SVG detection.

To evaluate its effectiveness, we tested MCFST on two simulated and three real datasets. On simulated data, MCFST showed superior robustness in domain recognition, outperforming advanced baselines. On real datasets, including human breast cancer tissue, it achieved higher clustering accuracy and more precise tissue delineation, while also identifying more SVGs with stronger spatial patterns than SpaGCN.

Compared with other multi-view methods such as SpaNCMG, which may suffer from view divergence due to large discrepancies across views, MCFST mitigates this problem through mutual information maximization and graph fusion, ensuring cross-view consistency and maintaining performance. Comprehensive tests confirmed that MCFST outperforms SpaNCMG in spatial domain recognition across both simulated and real datasets.

Although MCFST demonstrates strong performance in spatial domain identification and SVG detection, several limitations remain. First, the construction of multiple views in MCFST depends on predefined graph-building strategies, such as KNN-based spatial adjacency and expression-similarity graphs. Although these views provide complementary information, the optimal selection and weighting of views may vary across tissues, platforms, and data resolutions. Future work will explore more adaptive view construction strategies to automatically identify informative graph structures from spatial transcriptomics data. Second, the current framework mainly focuses on gene expression and spatial coordinate information, while histological images and other multimodal information are not fully incorporated. Integrating image-derived morphological features may further improve the ability of MCFST to characterize complex tissue architectures. Third, although MCFST performs well on the evaluated datasets, its computational efficiency and scalability should be further improved for larger and higher-resolution spatial transcriptomics datasets. Future studies will optimize the graph construction and model training process to reduce computational costs. In addition, further validation across more tissue types, disease contexts, and spatial transcriptomics platforms will be important to assess the generalizability of MCFST. These directions may further enhance the robustness, interpretability, and applicability of MCFST in spatial transcriptomics analysis.

## 4 Materials and methods

### 4.1 Ablation study

We used spatial transcriptomics datasets from the 10X Visium platform as baselines, including human brain, human breast cancer, and mouse brain tissues, and further tested on simulated datasets derived from the human breast cancer data. The human dorsolateral prefrontal cortex (DLPFC) dataset contains 12 slices, each with 5–7 cortical layers (Maynard *et al*. 2021); four slices (151509, 151510, 151671, 151672) were selected for experiments. The human breast cancer dataset includes four tissue categories (e.g. DCIS/LCIS and healthy tissue) manually annotated into 20 regions. The mouse anterior brain dataset was annotated into 52 regions across sections. Two types of simulated datasets were constructed from the breast cancer data: (1) Sparsity simulation: randomly masking elements in the expression matrix at ratios 0–0.8 (e.g. 0.8 means 80% entries set to zero). (2) Noise simulation: introducing Poisson-distributed noise at levels of 10%, 30%, 50%, 70%, and 90%.

### 4.2 Data preprocessing

We mainly used the tool Scanpy (Wolf *et al*. 2018) for data preprocessing of the dataset used. First, genes expressed in fewer than five cells were filtered out, after which the expression matrix was subjected to normalization and log transformation. In the second step, we selected the top 2000 and 3000 highly variable genes and rescaled the dataset accordingly. As the final step of PCA was performed on the expression profiles of the 3000 most variable genes, compressing them into 1000 dimensions. The resulting low-dimensional representations were subsequently adopted as feature vectors to characterize each individual spot.

### 4.3 Construction of multiple views

MCFST constructs four graph branches from three types of complementary information, including spatial adjacency, gene expression similarity, and spatially enhanced gene expression similarity. Specifically, two branches are constructed based on the same spatial adjacency matrix derived from spot coordinates, while the other two branches are constructed from gene expression similarity and spatially enhanced gene expression similarity, respectively. Although the two spatial adjacency branches share the same graph topology, they serve different functions during training. The first spatial branch is used as a basic spatial structural view for multi-view graph convolution, enabling local information propagation among spatially neighboring spots. The second spatial branch is introduced as an additional spatial-prior branch in the multi-view fusion framework, which reinforces the spatial consistency constraint during representation learning and graph fusion. In this way, the duplicated spatial adjacency is not intended to provide a new topology, but to strengthen the influence of spatial neighborhood information when integrating heterogeneous views.

For the construction of the spatial adjacency matrix, the KNN algorithm is applied to identify the 10 closest neighbors of each spot based on spatial location coordinates, which are then utilized to build the corresponding two adjacency matrices *A^(1)^* and *A^(2)^*, and the two identical spatial adjacency maps *G^(1)^*and *G^(2)^* are constructed using these two adjacency matrices. Taking *A^(1)^* as an example, *A^(1)^_ij_*= *A^(1)^_ji_* =1 is set if spot *i* and spot *j* are the nearest neighbors, otherwise it is set to 0. And, using the KNN algorithm to select the first 3 and the first 8 nearest neighbors of the spots using the spatial location coordinates as inputs to the mutual information maximization module, respectively. These two neighborhood sizes were used to capture spatial consistency at different local scales: the top 3 nearest neighbors provide highly local and reliable spatial constraints, while the top 8 nearest neighbors introduce a broader local context and increase the diversity of positive pairs. This setting helps balance local spatial smoothness and neighborhood diversity in the mutual information maximization process.

In the construction of spatially enhanced gene expression similarity map, follow the MNMST algorithm to construct cell expression network, use the spatial location information of spot to enhance the gene expression data of spot, use self-representation learning (SRL) to construct the adjacency matrix *A^(3)^* and use this adjacency matrix to construct the spatially enhanced gene expression similarity map *G^(3)^*. If spot i and spot j are identified as nearest neighbors, then set $A_{ij}^{\mathrm{(3)}}$= $A_{ji}^{\mathrm{(3)}}$=1, otherwise set it to 0.

For the construction of the gene expression similarity graph, PCA was applied only for graph construction. Specifically, the top 2000 highly variable genes were reduced to 50 dimensions, and the resulting 50-dimensional representations were used to calculate expression similarity among spots. Based on these representations, the KNN algorithm with k = 10 was used to identify the nearest neighbors of each spot and construct the neighbor-joining matrix *A^(4)^*, and this neighbor-joining matrix is used to construct the gene expression similarity graph *G^(4)^*. Similarly, if spot i and spot j are nearest neighbors, then set $A_{ij}^{\mathrm{(4)}}$= $A_{ji}^{\mathrm{(4)}}$=1, otherwise set it to 0. It should be noted that these 50-dimensional representations were used solely for graph construction, whereas the model input features were obtained separately from the top 3000 highly variable genes reduced to 1000 dimensions by PCA in the data preprocessing step.

### 4.4 Multi-view graph convolutional network model

The MCFST framework is composed of three main components: a multi-view graph convolutional autoencoder and a module for mutual information maximization, and a graph fusion network. The graph fusion network integrates four types of views, including spatial neighbor graphs, to construct a consistency graph that guides the autoencoder in learning unified representations. The multi-view graph convolutional autoencoder, together with the mutual information maximization module, is designed to learn low-dimensional graph embeddings of each spot from four complementary views, including spatial adjacency graphs. These embeddings are then aligned through a consistency graph generated by the graph fusion network, which ultimately yields a unified clustering representation.

#### 4.4.1 Multi-view graph convolutional network model

In order to consistently integrate different neighbourhoods contained in different views, a graph fusion network is used to generate a consistent graph G*. To fuse each graph, the first layer in the graph fusion network is a multi-graph fusion layer defined as:


(#(1)) G(1)=σ(∑m=1MWf(Wg(1)mG(m)+bg(1)(m)))

Where σ denotes the activation function, *W_f_* is the matrix of attention coefficients for the importance of edges in different views, *M* is the number of views (*M = 4*), *G^(m)^* denotes the *m-th* view, and $W_{g(1)}^{m}$ and $b_{g(1)}^{\left(m\right)}$ represent the weight matrices and bias terms corresponding to the different views in the first layer of the graph fusion network, respectively.

The consistency graph learnt at layer l in the graph fusion network is:


(#(2)) G(l)=σ(Wg(l)G(l−1)+bg(l)) 

Where $W_{g(l)}$ denotes the weight matrix, $b_{g(l)}$ represents the bias associated with the *l-th* layer in the graph fusion network, respectively and σ denotes the activation function.

The loss function of the graph fusion network is formulated as:


(#(3)) LG=∑m=1Mloss(G(m),G*)

Where $\mathrm{loss}(G^{\left(m\right)},G^{*})$ denotes the reconstruction loss of the *m-th* in relation to the consensus graph $G^{*}$.

ReLU is used in the hidden graph convolutional layers, while an identity activation is adopted in the final graph convolutional layer.

#### 4.4.2 Multi-view autoencoder

In a multi-view self-encoder, the first layer consists of a graph convolution layer corresponding to each of the m views, and the inputs to the first layer are the gene expression feature matrix *X* of the spot and the graph $G^{\left(m\right)}$ corresponding to each view. Subsequently, the embedding representation $Z_{\left(1\right)}^{\left(m\right)}$ corresponding to the *m-th* view, as learned through the first layer, can be derived using the following equation:


(#(4)) Z(1)(m)=σ((D(m))-12G(m)′(D(m))-12XW(1)(m))

Where σ denotes the activation function, which is set to ReLU in this study, ${G^{\left(m\right)}}^{\prime }$= $G^{\left(m\right)}$ + *IN* represents the correlation coefficient matrix after adding self-connections, IN is the unit matrix, ${D^{\left(m\right)}}_{ii}=\sum_{j}{G_{m}\prime }_{ij}$, $W_{\left(1\right)}^{\left(m\right)}$ refers to the learnable parameter matrix corresponding to the *m-th* view in the first layer.

In order to integrate the embedded representations of all views, the embedded representations of the spot in different views are fused using a multi-view fusion layer, and the multi-view fusion layer obtains the common embedded representation $Z_{\left(2\right)}$ with the formula:


(#(5)) Z(2)=σ(∑m=1M βm((D(m))-12G(m)′(D(m))-12Z(1)(m)W(2)(m)))

Where $\beta_{m}$ is a hyperparameter indicating the weight of each view, $\sum_{m=1}^{M}\beta_{m}=1$, $W_{\left(2\right)}^{\left(m\right)}\;$refers to the learnable parameter matrix associated with the *m-th* view in the second layer and σ denotes the activation function, which is set to ReLU in this study.

Finally, the graph convolution layer generates the unified embedding representation Z:


(#(6)) Z=σ((D*)-12G*′(D*)-12Z(2)W(3))

Where ${D^{*}}_{ii}=\sum_{j}{{G^{*}}^{\prime }}_{ij}$, $W_{\left(3\right)}$ is the trainable parameter matrix and σ denotes the activation function, which is set to ReLU in this study.

In order to enable the multi-view autoencoder to capture a unified embedding representation across different views *Z*, a graph decoder is employed. This decoder reconstructs the graph structures of each individual view by utilizing the shared embedding representation, denoted as ${\hat{G}}^{\left(1\right)}$, …, ${\hat{G}}^{\left(m\right)}$. Since the learnt common embedding representation already contains the spot features and the graph structure information, an inner-product decoder is used to predict the connectivity relationship between the spots in the graph structure, and the formula for reconstructing the graph structure of a view is given as:


(#(7)) G^(m)=sigmoid(Z·W(m)·(Z·W(m))T)

Where $W^{\left(m\right)}$ denotes the learned parameter matrix of the *m-th* view.

The multi-view autoencoder is trained by minimizing the sum of reconstruction errors across all views, computed as the difference between $G^{\left(m\right)}$ and its reconstruction ${\hat{G}}^{\left(m\right)}$:


(#(8)) Lrec=∑m=1MLrec(m)=∑m=1Mloss(G(m),G^(m))

Here, $L_{\mathrm{rec}}^{\left(m\right)}$ represents the reconstruction loss associated with the *m-th* view, while $L_{\mathrm{rec}}$ refers to the total reconstruction loss obtained by summing across all views.

Mutual information, derived from Shannon entropy, quantifies dependency between random variables (Belghazi *et al*. 2018). In deep learning, maximizing mutual information encourages models to learn meaningful representations (Bachman *et al*. 2019). Moreover, if two samples are similar, their low-dimensional embeddings should remain close in latent space (Wen *et al*. 2019). In spatial transcriptomics, this principle implies that adjacent spots with similar gene expression profiles should also have similar embedded representations.

Based on this assumption, we introduce a mutual information maximization module to enhance representation consistency among neighboring spots. Specifically, the embedding representation of a spot and that of its neighboring spot are treated as a positive pair sampled from the joint distribution, whereas the embedding representation of the spot and a randomly shuffled spot are treated as a negative pair sampled from the product of marginal distributions. A discriminator D(⋅,⋅), implemented by fully connected layers with Sigmoid activation, is used to distinguish positive pairs from negative pairs.

In this study, the mutual information maximization module adopts a Jensen–Shannon divergence (JSD)-based mutual information lower-bound estimator. The objective can be formulated as:


(#(9)) IJSD=1NK∑n=1N∑k=1K[log⁡ D(zn,znk)+log⁡(1-D(zn,z∼n))]

Where $z_{n}$ denotes the embedding of the n-th spot, $z_{n_{k}}$ denotes the embedding of its k-th neighboring spot, and ${\tilde{z}}_{n}$ denotes the embedding of a randomly shuffled spot. Maximizing this objective increases the agreement between neighboring spots while reducing the agreement between unrelated spots. Therefore, the loss function used in model training is the negative form of this lower-bound objective:


(#(10)) Lmim=-1NK∑n=1N∑k=1K[log⁡Rnk+log⁡1Fnk]

where $R_{nk}=D(z_{n},z_{n_{k}})$ is the discriminator output for positive pairs, and $F_{nk}=D(z_{n},{\tilde{z}}_{n})$ is the discriminator output for negative pairs. By minimizing $L_{\mathrm{mim}}$, MCFST maximizes the JSD-based mutual information lower bound between neighboring spot representations, thereby improving cross-view representation consistency.

Moreover, model optimization is performed by minimizing the discrepancy between the original probability distribution *Q* and the target probability distribution *P*, both defined over the shared embedding representation *Z*. First, if the number of spatial domains is known, the K-Means clustering algorithm is used to initialize the cluster centre *µ_j_* using the consistent embedding *Z*, where *µ_j_* (*j = 1, …, k*) denotes the centre of the *j-th* cluster, but when the number of spatial domains is unknown, the cluster centres are initialized using the Louvain clustering algorithm. Secondly, a t-distribution is used to compute the similarity between the embedding *z_i_* and the clustering centre *µ_j_*. Thus, the original probability distribution *Q* of the embedding representation *Z* is formulated as follows:


(#(11)) qij=(1+||zi-μj||2)-1∑j′(1+||zi-μj′||2)-1

Where *q_ij_* is the probability that node *i* is assigned to cluster *j*.

Subsequently, we calculate the target probability distribution *P* corresponding to *Z*. where each element *p_ij_* satisfies 0≤ *p_ij_* ≤1. The value of *p_ij_* is calculated as follows:


(#(12)) pij=qij2fi∑j′qij′2fj′

Where $f_{i}=\sum_{i}q_{ij}$ is the soft clustering frequency.

The probability distribution loss is calculated as follows:


(#(13)) Lpd=||Q-P||F2 

Therefore, the total objective function LM of the multi-view autoencoder is defined as:


(#(14)) LM=Lrec+λ1Lmim+λ2Lpd

Where *λ_1_* and *λ_2_* are hyperparameters that balance these three loss functions.

#### 4.4.3 Clustering

After learning the common embedding representation, if the spatial transcriptome dataset has provided the number of spatial domains, the K-Means clustering algorithm (Arthur and Vassilvitskii 2007) is employed to determine the spatial domains. When the number of domains is not specified in a spatial transcriptomic dataset, spatial domains are instead determined using the Louvain clustering algorithm implemented in Scanpy.

#### 4.4.4 Spatially variable gene identification

Following clustering, spatially variable genes (SVGs) were identified with reference to the detected domains, following the strategy of SpaGCN. Differential expression analysis was performed between spots within a target domain and those in adjacent domains using the Wilcoxon rank-sum test. To ensure the reliability of the identified SVGs, we applied the default filtering criteria used in our implementation. Specifically, genes were retained only when they satisfied the following thresholds: adjusted *P*value < 0.05, in-group fraction > 0.8, in/out-group ratio > 1, and fold change > 1.9. These filtering criteria were used to ensure that the selected SVGs were not only statistically significant but also exhibited clear and robust spatial expression patterns.

### 4.5 Evaluation criteria and baseline methodology

#### 4.5.1 Evaluation criteria

To assess the performance of spatial domain identification, we adopt the ARI (Rand 1971) is used as an evaluation metric. The ARI is computed as follows:


(#(15)) ARI=∑i∑j(nij2)-∑i(ai2)∑j(bj2)(n2)∑i(ai2)+∑j(bj2)2-∑i(ai2)∑j(bj2)(n2)

Where *a_i_* represents the number of samples assigned to the *i-th* predicted cluster, while *b_j_* corresponds to the number of samples included in the *j-th* true cluster. The term *n_ij_* specifies the count of samples shared in common between these two clusters.

To evaluate the spatial significance of identified SVGs, we adopted two classical spatial autocorrelation indices: Moran’s I and Geary’s C. Their definitions are:


(#(16)) Moran′s I=NW∑i∑j[wij(xi-x¯)(xj-x¯)]∑i(xi-x¯)2

(#(17)) Geary′s C=N2W∑i∑j[wij(xi-xj)2]∑i(xi-x¯)2

Where *x_i_* and *x_j_* denote the expression levels of a given gene at spots *i* and spot *j*, respectively, while $\bar{x}$ denotes the average expression of that gene across all spots. The variable *N* indicates the total number of spots in the dataset. The term *w_ij_* corresponds to the spatial weight assigned between spots *i* and spot *j* based on their relative coordinates, and *W* represents the cumulative sum of all such weights. To construct the spatial weight matrix, for each spot we select its five closest neighbors according to spatial coordinates; if spot *j* falls within this set, then *w_ij_ = 1*, otherwise it is set to zero.

Geary’s C takes values in the range [0,2]. For consistency with Moran’s *I* (range [−1,1]), we report 1-Geary’s C. Thus, values near 1 indicate strong positive autocorrelation (similar values cluster spatially), 0 indicates no autocorrelation (random distribution), and –1 indicates negative autocorrelation (similar values are spatially distant). For each gene, Moran’s I and 1-Geary’s C values generally align.

#### 4.5.2 Baseline methodology

In order to assess the capability of the proposed MCFST algorithm for accurate spatial domain recognition, MCFST is compared with SEDR, BayesSpace, SpaGCN, SpaNCMG, GraphST and MNMST. Moreover, in the comparative experiments for spatial domain recognition, all baseline algorithms were tested under their default settings, including parameter configurations, data preprocessing procedures, and clustering strategies. In addition, the spatially variable gene recognition performance of MCFST and SpaGCN was compared using the same spatially variable gene screening criteria.

## Data Availability

The spatial transcriptomics datasets used in this study are publicly available: (1) 10x Visium human breast cancer dataset: https://www.10xgenomics.com/resources/datasets; (2) 10x Visium human dorsolateral prefrontal cortex dataset: http://spatial.libd.org/spatialLIBD/; (3) The mouse brain anterior section dataset: https://www.10xgenomics.com/resources/datasets.
